# Internet of Medical Things: An Effective and Fully Automatic IoT Approach Using Deep Learning and Fine-Tuning to Lung CT Segmentation

**DOI:** 10.3390/s20236711

**Published:** 2020-11-24

**Authors:** Luís Fabrício de Freitas Souza, Iágson Carlos Lima Silva, Adriell Gomes Marques, Francisco Hércules dos S. Silva, Virgínia Xavier Nunes, Mohammad Mehedi Hassan, Victor Hugo C. de Albuquerque, Pedro P. Rebouças Filho

**Affiliations:** 1 Department of Computer Science, Federal Institute of Education, Science and Technology of Ceará, Fortaleza CE 60040-215, Brazil; fabricio.freitas@lapisco.ifce.edu.br (L.F.d.F.S.); iagsoncarlos@lapisco.ifce.edu.br (I.C.L.S.); adriell.gomes@lapisco.ifce.edu.br (A.G.M.); herculessilva@lapisco.ifce.edu.br (F.H.d.S.S.); virginia.nunes@lapisco.ifce.edu.br (V.X.N.); victor.albuquerque@ieee.org (V.H.C.d.A.); pedrosarf@ifce.edu.br (P.P.R.F.); 2 Department of Teleinformatics Engineering, Federal University of Ceará, Fortaleza CE 60020-181, Brazil; 3 Information Systems Department, College of Computer and Information Sciences, King Saud University, Riyadh 11543, Saudi Arabia

**Keywords:** deep learning, mask R-CNN, fine-tuning, transfer learning, Image lung segmentation

## Abstract

Several pathologies have a direct impact on society, causing public health problems. Pulmonary diseases such as Chronic obstructive pulmonary disease (COPD) are already the third leading cause of death in the world, leaving tuberculosis at ninth with 1.7 million deaths and over 10.4 million new occurrences. The detection of lung regions in images is a classic medical challenge. Studies show that computational methods contribute significantly to the medical diagnosis of lung pathologies by Computerized Tomography (CT), as well as through Internet of Things (IoT) methods based in the context on the health of things. The present work proposes a new model based on IoT for classification and segmentation of pulmonary CT images, applying the transfer learning technique in deep learning methods combined with Parzen’s probability density. The proposed model uses an Application Programming Interface (API) based on the Internet of Medical Things to classify lung images. The approach was very effective, with results above 98% accuracy for classification in pulmonary images. Then the model proceeds to the lung segmentation stage using the Mask R-CNN network to create a pulmonary map and use fine-tuning to find the pulmonary borders on the CT image. The experiment was a success, the proposed method performed better than other works in the literature, reaching high segmentation metrics values such as accuracy of 98.34%. Besides reaching 5.43 s in segmentation time and overcoming other transfer learning models, our methodology stands out among the others because it is fully automatic. The proposed approach has simplified the segmentation process using transfer learning. It has introduced a faster and more effective method for better-performing lung segmentation, making our model fully automatic and robust.

## 1. Introduction

The principal exam for obtaining thoracic medical images, which allows a clear view of the patient’s pulmonary airways, is the Computed Tomography (CT) [1]. CT is a high-contrast exam of the thoracic region of pulmonary structures and anatomy. CT is efficient for different medical image analyses; the examination gathers relevant information in the image through the density of pixels. Thus an improved analysis of the structures of patients and organs is possible [2]. Through CT scan, is possible to identify and diagnose various diseases by their shape or texture, such as Pulmonary Emphysema, Pulmonary Fibrosis, Lung Cancer and other Chronic obstructive lung disease in a non-invasive, effective, and fast way [3].

The detection of abnormalities in CT scans is of great importance for preventing diseases detected at an early stage: conditions such as lung cancer, if diagnosed in advance, can be better treated, which significantly increases the chances of recovery and decreases the mortality index [4]. About 65 million people have chronic obstructive pulmonary diseases and approximately 3 million of these people die each year from these diseases. In 2010, it was the world’s third leading cause of death [5]. According to the World Health Organization, in 2018, about 10 million people worldwide contracted tuberculosis [6]. It was estimated an incidence of 2.09 million new lung cancer cases, with 1.76 million deaths [7]. The first step in diagnosing a pulmonary pathology is the lung segmentation in CT images [8], which are performed by medical specialists. They manually mark the region of interest to perform analysis regarding the type of pathology and its aggravation, and thus arrive at a definitive diagnosis [9].

However, this diagnostic procedure is subject to a series of errors and uncertainties, which may cause inaccuracy of the diagnosis [10]. Bechtold et al. [11] carried out a study to analyze the factors that influence the error rate of the interpretation of abdominal computed tomography scans in an academic medical center. This study had 694 patients with an average age of 54 years, whose abdominal CTs were analyzed by three to five university radiologists. It was found that the margin of errors was correlated with the reader’s viability, the number of cases reads per day, the presence of a resident, the patient’s situation as being external or internal to the institution, and their genetic conditions. Among the results obtained, the reports recorded 56 errors in 53 patients, which led to a general error rate of 7.6%. Besides, 19 of these errors were considered clinically significant, and seven directly affected the patient’s management [10,11]. Aware of the difficulties in diagnosing lung diseases, several researchers have joined forces to elaborate and construct tools to assist medical specialists in the most diverse operations. For instance, they can help in the detection of diseases in exams [12], in the segmentation of whole organs [13] for a better analysis of a specialist, or even in the segmentation of the regions visibly affected by the disease [14]. These tools are known as Computed Aided Diagnosis (CAD) [15].

Several pieces of research have been developed to aid medical diagnosis through digital image processing, machine learning [16], and deep learning [17]. For instance, Pehrson et al. [18] proposed a general study on Machine and Deep Learning techniques applied to a database of lung images available in the literature, such as the detection of pulmonary nodules in CT images. Their study was carried out through extensive research of 1972 publications in total, of which 41 were selected to compose the work. Most of the techniques obtained accuracy equal to or greater than 90% when compared to the ground truth provided by the specialist doctor.

New approaches involving transfer learning with methods such as fine-tuning provide better results for the segmentation of objects contained in an image, such as the region of interest in medical images [13]. The works of Shojaii et al. [19] present the importance of segmenting the pulmonary airways as a fundamental step to aid diagnosis using medical images. After lung segmentation, it becomes possible to analyze the airways [20], density [21], or diaphragm [22]. One of the methods currently used for medical image segmentation is Convolutional Neural Networks (CNN) [23,24]. The Parzen-window method consists of a nonparametric model to estimate a probability density function. Parzen-window, used as a segmentation method, estimates the probability of a pixel relating or not a given domain, making it easier to view the object of interest contained in the image [25]. Parzen-window can be used as a fine-tuning tool to improve Machine and Deep Learning techniques if used well [26].

An example of efficient use of CNN to assist the medical diagnosis of lung diseases is the work by Lin et al. [27]. They developed a 2D CNN study with parametric optimization Taguchi [28] to recognize lung cancer from CT scans automatically. The authors used 36 experiments and eight mixed-level control factors to select the ideal parameters for the 2D CNN. The method obtained accuracy between 90% and 98% according to the datasets used.

IoT has become an increasingly recurring reality in the most varied technology and services branches due to the emergence of new technologies and higher communication speed between devices. These applications are in areas such as robotics [29], security [30], and even health: The so-called Personalized Healthcare (PH) is a new approach to healthcare in the field of monitoring. The service uses patient data through health records, sensor signals, wearable devices, web-based information, and social media, among others. Machine Learning techniques and artificial intelligence are applied to this data set to generate analytics. Then, specialists study this information to plan a better lifestyle and disease prevention for their patients [31].

The focus of these new advances is patient data collected from electronic health records (EHR), IoT sensing devices, wearable and mobile devices, web-based information, and social media. The PH applies Artificial Intelligence (AI) techniques to the data set collected to improve techniques of disease progression, disease prediction, patient self-management, and clinical intervention. Tools with the use of IoT in the health area are increasingly explored in recent studies. The use of these tools frequently assists medical diagnoses, such as the approach by Tao Han et al. [32], which used IoT to detect lung and brain in CT images. The authors used a combination of networks and achieved good results with fine-tuning. The use of segmentation in medical images in lung CT scans is a complex challenge in image processing, as shown by the work of Souza et al. [33] that used techniques based on Mask R-CNN for segmentation of pulmonary regions in CTs. There are several studies in the area of the Internet of Medical Things, as shown by et al. Arthur Gatouillat [34]. They addressed techniques and studies related to IoT in the field of medicine and several solutions.

These powerful techniques for diverse existing lung diseases, in general, have something in common: good lung segmentation to make the application of disease detection optimized. Good pulmonary segmentation can optimize the method used to dispense with auxiliary fine-tuning techniques, for example [35], who used CNN to target lung regions. The same occurred with the study by Souza et al. [36], which used the CNN Mask R-CNN network to segment lung images on CTs. The results were excellent with accuracy above 97%, raising the quality level of many studies using deep learning. The segmentation problem in medical images is classic and challenging. Several methods of Digital Image Processing (DIP) bring solutions in a non-automatic way, which through various combinations of image processing, reach satisfactory results. Taking into account all pre-processing and processing in order to achieve levels of quality in its results, techniques such as these are very relevant in the post-processing of deep learning networks. The combinations of classic methods in the post-result generated by networks bring great improvements through fine-tuning methods, making the results more relevant and meaningful for processing with deep processing networks.

Given the importance of pulmonary segmentation in computed tomography images, either for the process of assisting the diagnosis or assisting with the accompaniment of the pathology. The model proposed in this study uses a new approach with fine-tuning Convolutional Neural Network architecture (R-CNN mask) aided by Parzen-window method [37]. Also, into account the demand for increasingly practical and accessible systems for the most varied services. The system developed in this study has a tool that can be accessed remotely by devices with internet use.

The proposed work aims to study the following thematic areas within the research:

- The application of computer vision tools based on IoT in medical images.
- The use of deep extractors for the classification of pulmonary CT images.
- CT segmentation of lung images based on deep learning techniques.
- The use of Deep Learning with a fine-tuning technique based on Mask R-CNN and Parzen-window.

## 2. Related Works

Different computational methods are applied to medical images for different problems searching for solutions to segment objects in medical images [17]. Different techniques address the use of filters based on detection and image segmentation. The lung is segmented using the Digital Contour Processing transformation technique, as shown in the work [38], which is based on a detection technique to smooth out the extracted lungs contours, showing efficiency by achieving an average Jaccard result of 94%. Thus, different segmentation methods have been proposed to aid medical diagnosis [39] to detect lung diseases such as cancerous nodules [40], cystic fibrosis [41] or detect lung physiological structures [42].

However, in the face of a large part of the more complex problems, Digital Image Processing techniques are inefficient, with results below expectations. It is then necessary to use more advanced techniques to solve the proposed problems. With the evolution of hardware processing capabilities, deep learning techniques have been more explored [43] and consequently used for the segmentation of physiological structures in medical images [44]. Wang [45], proposed the use of Convolution Neural Networks to segment pulmonary nodules, the method obtained results around 80% Accuracy, which means a good ratio of targeted hits to total pixels in the image. But showed difficulty in segmenting smaller nodules. Duraisamy [46] developed a segmentation method using techniques of DIP and Convolutional Neural Networks, applied in Magnetic Resonance Imaging (MRI) on brain and chest images exams.

When proposing machine learning methods with medical images, a specific and constant problem is the absence of labeled data to develop proper model training. Several works use transfer learning and fine-tuning methods to provide proportions of solutions for medical images and successively improve their results [47]. Fine-tuning allows a network model that has already been trained for a specified task to perform a second similar task. A network already designed and trained takes advantage of the resource extraction that occurs in the front layers of the network without developing this resource extraction network from scratch [48]. The work [49], proposes a new structure using Transfer Learning, allows CNN training to locate the lesion in breast regions. The method achieved 83% accuracy at its best.

Rebouças et al. [50] proposed an adaptive method based on Region Growth and Hydrographic Basin called Optimum Path Snakes (OPS) for the segmentation of CT images of Lung and brain. In their results, a Dice Coefficient of 93% was adopted, which proves the method’s effectiveness. However, it is noteworthy that the technique is based on digital image processing, having difficulty segmenting in non-standard cases. In this perspective, countless studies have been carried out [47], including Hu [13], who proposed the use of the Mask R-CNN network and machine learning for the segmentation of lung CT employing fine-tuning and transfer learning. The automatic network obtained good results with an accuracy of 97.68% and an average time of 11.2 s for segmentation. We use the same database to train our model and generate our results according to the proposed method.

With the advancement of communication between systems, the IoT has been spreading to various areas of knowledge. In the medical field, the IoT can implement support and monitor systems. Healthcare professionals who provide care, and even patients, can have remote access to sensor data collected from devices connected to people under monitoring or with special needs [51].

When placed in terms of CAD systems, the IoT can come in the form of an interface that directly connects the end-user to the tools for segmentation, detection, or classification of medical images. Thus, a device that initially needed the training to be used can be manipulated by any user through a visual interface [52]. Another application of IoT in conjunction with segmentation techniques is in the continuous monitoring of diseases, to measure their progression or regression. Aware of this application, Yao et al. [53] developed an IoT-based technique, along with fuzzy clustering techniques, Otsu thresholding, and morphological operations for the segmentation and monitoring of lung cancer in CT images, to make the prevention and monitoring of the disease progression easier and more efficient.

Han et al. [32] proposed a technique based on the health of things to perform the classification and segmentation of computed tomography images of the lung and hemorrhagic stroke. The IoT used in this work took the form of a tool for direct interaction with the user, which directly selects the best set of extractors and classifiers for the proposed problem. The authors were successful in their task of classifying and segmenting, with an accuracy above 95%.

With the help of the massive flow of medical data provided by the advancement of the IoT, Masood et al. [54] proposed the creation of a computer-aided decision support system to detect and classify lung cancer. Their technique is based on the deep learning model and information obtained from the Medical Body Area Network. The proposed model is called DFCNet, which is based on the deep convolutional neural network (FCNN) and detects pulmonary nodules in four different stages of cancer. The accuracy achieved with the FCNN was 84,58%, which shows the potential of the technique in helping radiologists detect nodules.

This work proposes a new lung segmentation method in CT images using the Mask R-CNN Model as initialization to apply the fine-tuning technique in the Parzen-window method. Thus the expected result is a segmentation with a high standard and with more speed. Besides, the technique is based on IoT to help select the best deep extractor/classifier combination to optimize the method and make it a practical and innovative tool to aid medical diagnosis. Finally, to validate our study, the results obtained were compared to the works of [13,50].

## 3. Background

### 3.1. LINDA

With the advancement of technology, the emergence of alternatives to aid medical diagnosis has been boosted and developed. Thus, through IoT, technologies and proposed methods are applied to achieve this objective. The present study uses the Lapisco Image Interface for the Development of Applications (LINDA) [52] as a microservice. This IoT system provides computer vision and signal processing resources and services for various cloud-hosted contexts. The API can communicate via the Representational State Transfer (REST) protocol through the JavaScript Object Notation (JSON). Through the use of advanced techniques of deep extraction and machine learning, it is possible to accurately evaluate CT images quickly to detect if there is a region of interest to start the lung segmentation stage.

**Figure 1:**
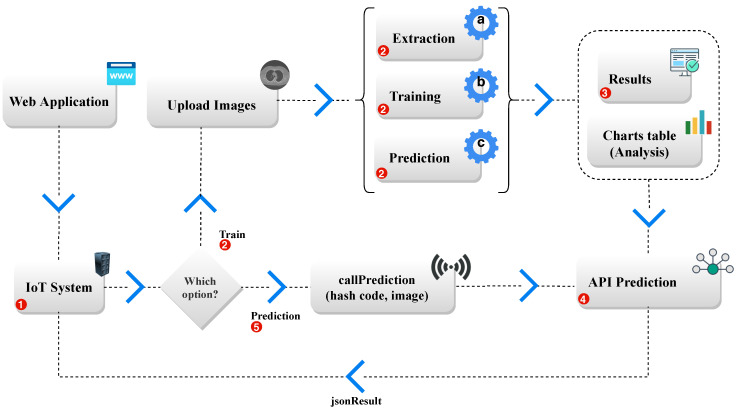
Illustration of the LINDA system architecture. The platform is accessed from computers, notebooks, and mobile devices. The IoT system (**1**) features a web access interface to select two options: training or prediction. In the training stage (**2**), the user must provide the image exams and the classes. Then, at (**2.a**), deep extraction of attributes is performed, generating a new one-dimensional vector data set. In (**2.b**), the training of classifiers is done, combining each extractor and classifier. In (**2.c**), the trained models perform a classification step in the test set previously separated. In (**3**), the results of experiments and its charts are made available, and the user can select the best combination. In (**4**), the model is made available through a cloud service that can be accessed from any device with Internet access. In the prediction stage (**5**), the user can access an API to perform a pre-diagnosis or, in the case of this work, classify the absence or presence of lung in the image. This figure represents STEP 1—IOT APPLICATION of Figure 3.

LINDA initially classified strokes only. However, with adjustments, the network can classify the presence or absence of any pathology in a medical image and what type of pathology is among the existing classes. The tool uses different CNNs with transfer Learning, combining it with the various classification techniques found in the literature. LINDA shows the result of these combinations to find the best one, proposing to be an efficient and flexible system to aid medical diagnosis, with a differential: its graphical interface allows its access from any computer or mobile device with internet access, even for users with little experience in Computer Vision [52]. The operation of the LINDA tool is illustrated in detail in the Figure 1.

### 3.2. Deep Learning Extractors

LINDA uses different CNNs to extract attributes from the set of medical images. Then, through transfer Learning, it applies the classification to the extracted attributes. These networks can extract attributes not reached by standard extractors, hence their importance. These CNNs are described below. The CNN Extreme Inception (Xception) is composed of Depthwise Separable Convolutions layers. The structure comprises 36 of these layers structured in a total of 14 modules [55]. MobileNet has 28 layers and is built in Depthwise Separable Convolutions layers (except the first layer [56]. The Oxford Visual Geometry Group (VGG) is a CNN that is of 11, 13, 16, or 19 layers; there is a positive correlation between increasing the models’ depth and accuracy in the ImageNet Dataset [57]. Inception-ResNet-V2 is composed by combining the Residual Connections with the model Inception [58]. The Dense Convolutional Network (DenseNet) makes shorter connections between adjacent layers connecting each layer to all others with the same input resolution [59]. The Residual Network (ResNet) introduced by Microsoft Research has a depth of up to 152 layers and has the standard input image size of 224 × 224 × 3 [60].

### 3.3. Classifiers

The classifiers used by LINDA in this study are: Bayesian classifier [61], Multilayer Perceptron (MLP) [62], k-Nearest Neighbors (KNN) [63], Random Forest [64], and Support Vector Machine (SVM) [65].

The Bayesian classifier uses the probability to classify the object as to the existing attributes. Thus, it does not express the sample class, but the probability for each class [61]. MLP is a classifier based on the joining of perceptrons, which is based on the organic neuron. MLP can solve nonlinear problems very effectively after a long training process [62]. The KNN is a classifier based on distances; the sample is classified according to the numerical distances of its attributes with the attributes of the samples already classified [63]. Random Forest is a conditional classifier that creates a sequence of decisions to classify the sample [64]. SVM is a non-probabilistic binary linear classifier, which, through regression, extends the sample class according to the trained pattern of the attributes [65].

### 3.4. Deep Learning

Mask R-CNN is a Convolutional Neural Network architecture based on deep learning that has the function of classifying the existing pixels in the image. This classification process involves the most varied layers used by the network, and vary according to the characteristics identified in the image. Its operating process consists of two stages: In the first, Mask detects the region of interest and demarcates it with a bounding box. In the second stage, the method consists of classifying the pixels of the demarcated region, in which, during the convolutional process of the network, the pixels selected as belonging to the region of interest are used for the construction of a mask. This mask will represent the detected region. The pixels that make up the region of interest are added to the mask using the formula:

(1) L=Lcls+Lbox+Lmask

In which Lcls represents the loss of the bounding box, Lbox represents the identical regions and Lmask represents the weight selected by the algorithm for a better selection of the pixels of interest [66].

**Figure 2:**
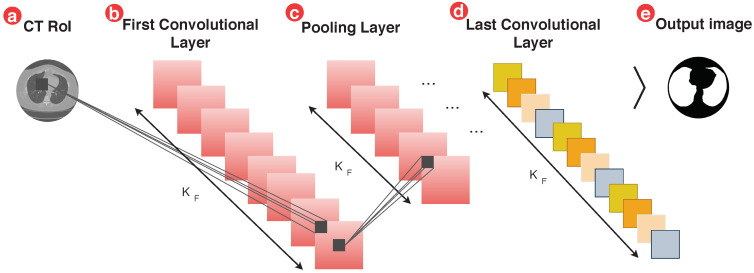
The ilustration of the deep features generation process on the CT images of the lung, in which the initial layers undergo a pooling process and only then form the last convolutional layer, this process makes the method less sensitive to the location of resources since the pooling method reduces sampling [67].

In item *a* of Figure 2, we have the input images. In item *b* we have the first convolutional layers. In deep models, these layers are stacked that allow the layers next to the input to learn low complexity through systematic filters learned from the input images. wherein item *c*, we have the pooling process that reduces the sampling along the spatial dimensions, reaching than the item *d*, which is responsible for processing high resources complexity level, and no item *e*, we have the segmented output image [67].

### 3.5. Parzen-Window

The Parzen window technique is a non-parametric function based on the calculation of probability density and pattern recognition. This method is very efficient for estimation and standards when there is no previous knowledge about the standards. The function aided by the weighted sum of some structuring element functions. It uses the sum of several distinct structuring elements, such as the Gaussian, uniform, and triangular structuring element. The Parzen window equation is given by:

(2) $$p\left(z\right)=\frac{1}{n}\sum_{i=1}^{n}\frac{\delta_{n}\left(z-z_{i}\right)}{hn}$$

In which p(z) is the probability of the pixel being or not in the region, where *z* corresponds to the pixel, δ is the structuring element function used to limit the neighborhood of the pixel, *h* is the size from the edge of the region, and *n* is the total number of pixels in the region [68].

The Parzen window function is used to lose part of its efficiency when it passes through areas of discontinuities in the estimation of the probability density function. To smooth out such discontinuities, the density model represented by:

(3) $$p\left(z\right)=\frac{1}{N{\left(\sqrt{2\pi }h\right)}^{D}}\sum_{n=1}^{N}exp[-\frac{1}{2}(\frac{∥x-x_{n}∥}{h}{)}^{2}]$$

where *h* now represents the density estimate of Gaussian components, *D* is n-dimensional Euclidean space, *N* represents the number of centered cubes, and the term $\left[\left(x-x_{n}\right)/h\right]$ will be a value of 1 if $x_{n}$ is within the region of interest in *z*, otherwise it will be a value of 0. Thus, the final value of the function results from the sum of contributions from several Gaussian models placed into a data set, and then it is normalized. The model becomes too sensitive to noise when the *h* is too small, while the loss of information occurs when *h* is too large [69].

### 3.6. Metrics

In order to validate our results, the following metrics were used: Accuracy, Sensitivity, Matthews, Dice, Jaccard and Hausdorff.

Accuracy is related to the number of pixels correctly segmented, with the total number of pixels segmented in the total [70].

Sensitivity, indicates how much pixels of the region of interest were correctly segmented among all the pixels in which they were segmented as part of this [70].

Matthews, represents the correlation of prediction of regions regardless of their size [71].

Dice, represents the similarity between segmented regions, this coefficient indicates how overlapped are the region segmented by the proposed method and the region obtained by the gold standard [72].

Jaccard, measures the similarity between two regions, according to the total number of pixels concatenated between both. In this way the segmented region is compared with the ground truth [73].

Hausdorff, which measures the degree of incompatibility between two sets, based on the calculation of distances between points of intersections present in the two regions [74].

Friedman test, the statistical test used in our experiments was the Friedman test, a non-parametric test that serves to assess whether a given distribution is similar or not to another distribution. Thus, one can indirectly affirm the superiority of a given distribution in relation to the other [75].

## 4. Methodology

### 4.1. Data-Set

The data set used by this work contains a set of 1265 lung CT images, in Digital Imaging and Communications in Medicine (DICOM) format, with dimensions of 512 × 512 pixels and 16-bit depth, containing the ground truth. The images were acquired in partnership with the Walter Cantídio Hospital of the Federal University of Ceará (UFC), Brazil, approved by the Research Ethics Committee. Committee–COMEPE (Protocol No. 35/06). We designed the experiments using 80% for training the model and 20% for testing.

### 4.2. Methodology

**Figure 3:**
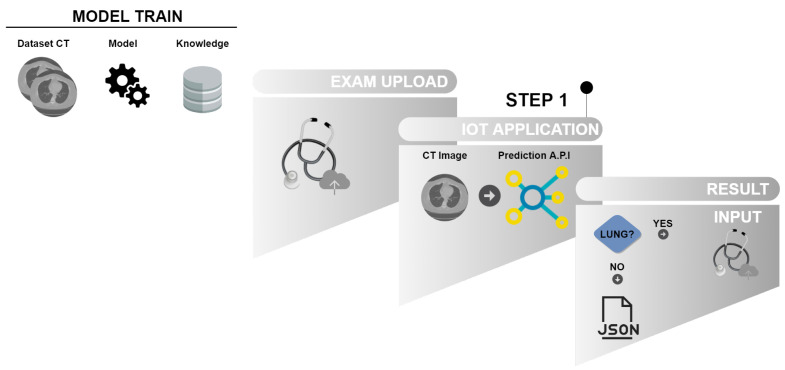
The API uses different CNNs and classifiers in an IoT-based architecture for the classification of lung images according to their presence or not in the other slices of the exam. The API uses a graphical interface for more significant interaction with the user, in which the user inserts the image bank and the classes to be classified. This interface then communicates with the server, which contains the algorithm (endpoint) that will apply the different CNN models used. Then, through Transfer Learning, it sends the attribute vectors to the classifiers used. This event co-occurs; each CNN model receives the same images and sends them to their respective classifiers. This process co-occurs, as there is a need to find the best combination of the CNN model for extraction and its respective classifier.

The methodology of this study is divided into two phases. The First phase, described in Figure 3, represents the classification for detection of lung images in CT scans using IoT, using trained models to identify the existence of lung in the input image of the proposed model. In this first phase, the LINDA [52] model detects pulmonary regions’ existence based on classic classifiers and classifiers based on deep learning to proceed to the second phase of the model, pulmonary segmentation phase.

**Figure 4:**
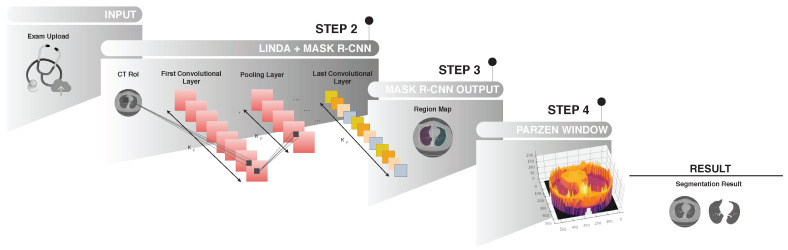
This Infogram represents the second phase of the proposed method. The image (INPUT), the result of the process of the first STEP 1 phase, is the input to the Mask R-CNN network. In (**STEP 2**), the model based on deep learning creates a map of the proposed region belonging to the lung represented by the image (**STEP 3**–Mask R-CNN OUTPUT). And finally, Transfer Learning process using Parzen’s Window method in (**STEP 4**) finishing with the segmentation of the lung region.

The second phase of the proposed model is shown in Figure 4. A lung map is created in Step 2, using the Mask R-CNN network to detect the lung region. After this process, the model uses fine-tuning to detect the edges of the lung region by readjusting it to the lung wall, as clarified in Steps 3 and 4.

#### 4.2.1. First Phase—Classification

Figure 3 illustrates the initialization of the model trained by the LINDA application [52]. After training the model, in this first phase, the image representation (EXAM UPLOAD) corresponds to the input images in the API for lung detection in CT images. It is in STEP 1, where APPLICATION IoT classifies the images of the model entry. If the API detects the lung in the image, the process proceeds to the Second Phase, as shown in Figure 3 in RESULT. If it does not find lung to be segmented on the CT image, the process is completed. In the First Phase, classifiers and extractors presented in Section 3 are used to detect pulmonary images on CTs.

In Figure 4, before starting the transfer learning process stages with our model for segmentation of lung CT, training of the model based on the R-CNN mask network is performed, designed to detect the area corresponding to the lungs. The set is trained based on the ground truth, as it uses only images already segmented by a specialist doctor. The knowledge generated by the Mask R-CNN model is stored at the end of the training.

#### 4.2.2. Second Phase—Segmentation

In Step 2, as shown in Figure 4, the model receives the image from the previous phase (Step 1) that contains the lung image. If the classifier does not find the lung in the CT image, the process already ends in phase 1 (Step 1) automatically. In the case of detection of the lung image, the model proceeds to Step 2. In (STEP 2), the Mask R-CNN network is used to create the lung map of the input image of the proposed model. After making the pulmonary map in (STEP 3), the fine-tuning process using the Parzen window in the (STEP 4) method using the Mask R-CNN network’s weights is followed. And finally, after STEP 4 with the result of pulmonary segmentation.

In STEP 2 of the model, the lung tomography image is processed with the R-CNN Convolutional Neural Net Mask. In the image (INPUT), the segmentation process based on our method is initiated with the insertion of the lung CT image in the model, process image from previous phase 1. In STEP 2 of the model, the lung tomography image processed with the Convolutional Neural Network Mask R-CNN, a network built in order to classify different types of objects with attributes stretched by their wide range of layers. The classification process is divided into two phases: In the first phase, the possible region of the lung’s existence is demarcated with a bounding box. During the second phase, the bounding box’s pixels are classified as belonging or not to the pulmonary regions. It is worth mentioning that the attributes used are selected based on the network architecture. Then, after the pixels are classified as belonging or not to the region of interest, the network performs the construction of the region of interest through the pixels classified as belonging to the pulmonary region.

After STEP 3 of the model, the Mask R-CNN classifier detects the region of interest parallel to its classification and detection process, applied over the lung tomography image pixels. The output of this classification is a demarcated (detected) region. This region, called a mask, is used in the second stage of operation of the proposed method: the mask can be considered as a kind of segmentation through the detection of the object, detailed its functioning in Section 3–A. However, the results are not entirely satisfactory, as the method captures by detecting the object as a kind of segmentation bypassing the object showing its location in the image.

In Step 4, to improve the detection contour for high-level segmentation, the result generated by Mask R-CNN has its dimensions reduced so that it can be used as an input parameter in the Parzen window function, being the starting point for the meeting of the edges of the lung. This decrease can be demonstrated in the Equation (1). The Parzen window function estimates the probability that a particular pixel belongs to a specific region or not. This estimation is performed by calculating a probability density function. The Gaussian kernel function smoothes the reallocation of the pixels marked as belonging to that region. The image generated by the Mask R-CNN is used as an input parameter for the Parzen function. The probabilistic calculation is performed on the image of the brain’s gray matter, more precisely in the Injured region.

Thus, with the overlay of the result generated by Mask-RCNN on the original image of the CT scan of the lung, the new segmentation region is formed. As shown in STEP 4, this segmentation region is stable, as it tends to grow or decrease its edges according to the results of the probability calculations performed by the Parzen window function, found in the Equation (2), and the main kernel function used is the Gaussian function, composed of the formula:

(4) $$\delta \left(z\right)=\frac{1}{\sqrt{{\left(2\pi \right)}^{n}\left|C\right|}}e^{-\frac{1}{2}{\left(z_{i}-z\right)}^{\prime }C^{-1}\left(z_{i}-z\right)}$$

where C, is the co-variance matrix *d* × *d* where *d* represents the dimension, |C| is the determinant of *C*, *z* and $z_{i}$ are the pixels.

The selected kernel function was Gaussian for two reasons. First, the Gaussian function is smoothed, and therefore the estimated density function p(z) also varies smoothly. The second reason, if a special form of the Gaussian family is assumed in which the function is radially symmetrical, the function can be specified completely only by a variation parameter. Thus, p(z) can be expressed as a mixture of radially symmetrical Gaussian cores with common variance δ [25].

**Figure 5:**
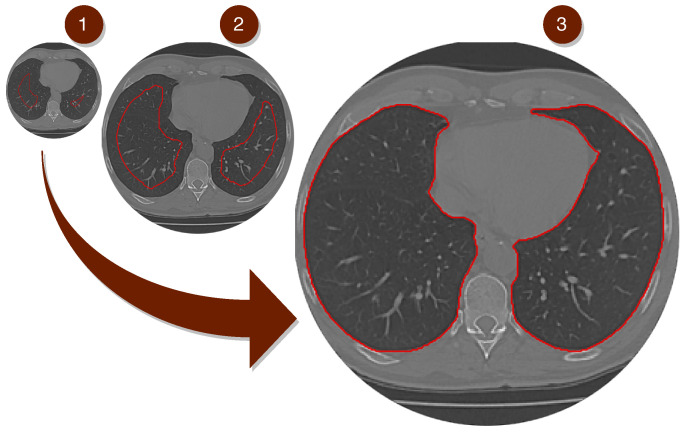
The Figure above shows the segmentation of the lung using our Composite Method, based on deep learning combined with the Parzen window method through Transfer Learning.

The Equations (4) direct the expansion to the edges of the lung, indicating which direction this movement should be in, as shown in the Figure 5.

In RESULT, after a series of expansions at the edges, the movement loses speed and energy, as the calculations stabilize. Then after fixing the edges of the lungs, the segmented region is extracted by concatenating the edges and their internal area, as shown in RESULT image.

**Figure 6:**
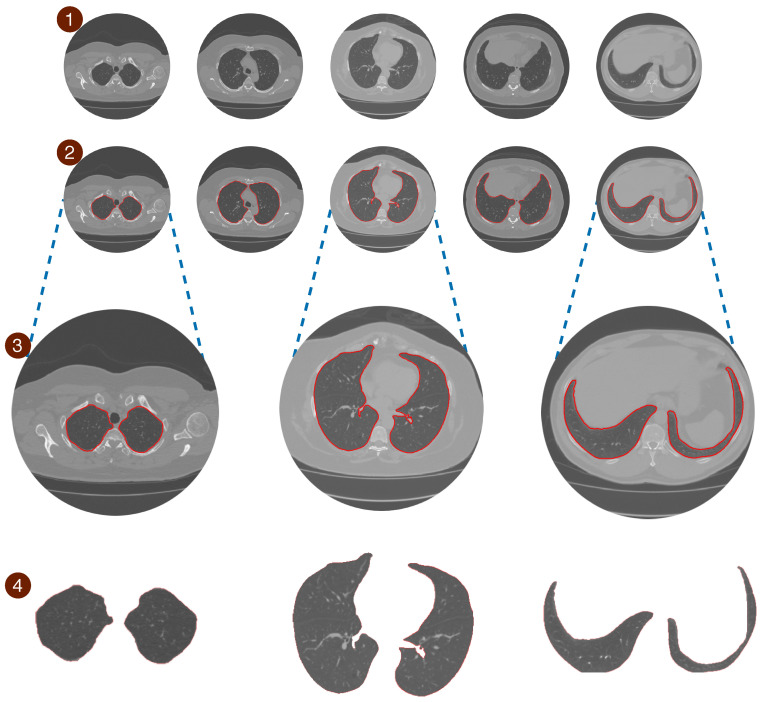
It presents the model’s segmentation differently; inline 1, the lung’s CT images are shown without segmentation methods. Inline 2, we offer the images already segmented by the proposed method. Inline 3, the images were enlarged for better visualization of the results. Inline four, the segmentation served as a basis for lung extraction for a better visual analysis of our model’s final result.

The pulmonary region can be obtained by subtracting the region segmented by the original image of the exam, as shown in the Figure 6, where it can be better analyzed visually.

## 5. Results and Discussion

The Results and Discussion Section the Experiment is divided into two Steps. In the First Stage, the result and discussion of the experiment is presented based on the classification of lung images using the IoT, where the API based on IoT identifies in the image shown at the entrance of the network, whether there are pulmonary regions or not. This First Stage represents the results generated by the classifiers in the detection of pulmonary regions through extractor combinations.

Next, the Second Stage of Results and Discussion consists of the results of the second stage of the experiment, as presented in Section 4 of Methodology, in which it represents the results of lung segmentation in CT images.

### 5.1. First Stage of the Experiment

This first stage of the experiment presents the result and discussion of the classifiers used in the model supported in this study (First phase of the Method). In the First phase, as presented in the Methodology Section 4, the trained and IoT-based model detects the existence of lungs in CT images. The model identifies the existence of the lung in the image to solve the problem of no lung in some CT images. This is because the human body’s process of inspiration and breathing of the lungs occurs naturally, inflating, and deflating the lungs during the CT examination.

Table 1 shows the results achieved by combining the deep attribute extractors InceptionResNetV2, Xception, NASNetMobile, DenseNet201, ResNet50, InceptionV3, VGG19, DenseNet169 and MobileNet with the classifiers Naive Bayes, MLP, Nearest Neighbors, Random Forest, and SVM with the Linear, Polynomial and RBF kernel. The metrics were obtained in the test set after training the classifiers to identify a lung or not in a CT image.

Initially, we normalized the training set by zero mean and standard deviation one, and the test set was also normalized using the same training set normalization rule. To optimize the classifier hyperparameters, we use the random search technique with 5-fold cross-validation. 80% for the training set and 20% for the validation set. The hyperparameters that reached the highest average of the correctness rates in the validation sets were chosen to solve the problem.

The intervals chosen for the classifier hyperparameters were: [2, 1001] neurons for the hidden MLP layer with the Adam optimizer, maximum of 4000 iterations or until the error reaches 1 and $10^{-3}$; 1,3,5,7,9 clusters for the KNN; the maximum number of decision trees for Random Forest was 1500; the SVM with Linear kernel had the *C* hyperparameter ranging from [$2^{-5},2^{15}$]; the degree of the SVM polynomial with the Polynomial kernel varied in the range 3,5,7,9 and *C* between [$2^{-5},2^{15}$]; lastly, SVM with RBF kernel had the *C* hyperparameter ranging from [$2^{-5},2^{15}$] and the γ hyperparameter ranging from [$2^{-15},2^{3}$].

We used the highest achieved value of the F1-Score metric as a selection criterion for the best model. The F1-Score is equivalent to a harmonic mean between the metrics of sensitivity and specificity. In the context of lung detection in CT images, the sensitivity denotes the model’s ability to detect lung in images that do present lungs. On the other hand, specificity indicates the model’s ability to classify images that do not present the lung region as non-lung, functioning as a complete sensitivity. Thus, the F1-Score metric seeks to reduce the rates of false negatives and false positives at the same time.

Thus, according to Table 1, the extractor/classifier combination that stood out in the test set was the Xception extractor with the MLP classifier, achieving the highest rate of F1-Score with 95.35%, demonstrating that this combination achieved the highest rates of sensitivity and specificity simultaneously. Also, the combination achieved the highest accuracy rate, with 98.42%. This indicates that Xception demonstrated greater capacity to extract representative characteristics to discriminate slices with lung and without lung. Thus, to detect the pulmonary region in CT images, we selected the Xception extractor with the MLP classifier for our approach. The purpose of this step is to reduce the exam’s full segmentation time since images that do not have a region of interest are not sent to the segmentation step. Also, filtering slices without lung contributes to improving the quality of the final segmentation result.

### 5.2. Second Stage of the Experiment

The Second Stage consists of the result generated by the second phase of this study’s proposed method, which is divided by Steps. In this second phase, the experiment aims to segmentation using the Mask R-CNN network to detect the whole region of the lung using lung maps, followed by fine-tuning techniques to find the edges of the lung region.

#### 5.2.1. Result Second Stage—(Step 1: Pulmonary Segmentation Using the Proposed Method)

This section is divided into two stages; the first stage corresponds to the experiment of the method proposed by this study. The second stage corresponds to the comparisons between other models and validation of the proposed method.

**Figure 7:**
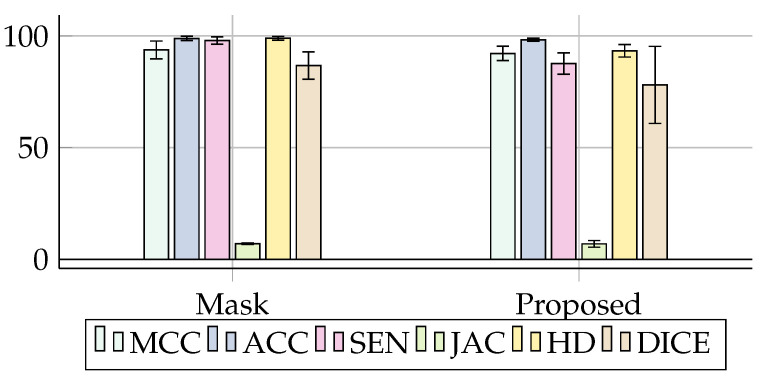
Illustration of the metrics obtained with the result of the Mask R-CNN against the output generated by the proposed Method.

In this first step, we used our model based on learning through fine-tuning, using the Mask R-CNN combined with Parzen’s window method as presented in Section 4. The segmentation results can be seen in Table 2 of this section, and presented visually by the Figure 7. The evaluation metrics used were (MCC, ACC, SEN, JAC, HD, DICE, TIME), briefly described in Section 3.

As can be seen, the model obtained an excellent result for segmentation of lung CT receiving ACC values in (98.34 ± 01.13), this means that among the methods used in the comparison method was the one that most correctly segmented the pixels as belonging to the Lung region among all the pixels in the image. This demonstrates the effectiveness of the method proposed by this study, where the model simplifies segmentation using the transfer learning technique to reuse the data generated by the deep learning network.

Bearing in mind that Mask R-CNN without transfer learning achieved in our experiments metric values of results lower than the model proposed with Accuracy (89.96 ± 04.38). It demonstrated that using the transfer of learning provides a better result for lung CT image segmentation. The model based on the transfer of the weights generated by the Mask R-CNN network adjusted to the Parzen method was superior in all the evaluation metrics presented in Table 2. With Mcc of (93.72 ± 03.99) against (58.03 ± 06.46), it is shown to be efficiently superior in the other metrics. This is due to the Mask R-CNN operation that detects the region of interest in the examination in a generalized way (bounding box) and then trims the edges. However, the Mask’s focus is not to segment but to detect the region of pulmonary interest. For a better result, it was necessary to use fine-tuning techniques based mainly on Parzen windows. The results were satisfactory because the Parzen window when receiving the detected Mask as an input image can calculate the edges of the region of interest effectively through its probabilistic estimation. Thus, the process of detection and segmentation of pulmonary areas through the transfer of learning demonstrated effectiveness in the results. The model was able to identify regions that are genuinely belonging to the lung effectively. This can be checked according to the effects of Accuracy and Sensitivity presented in the Table 2.

The proposed model’s notorious effectiveness can be seen in (Sen) and (Jaccard). Both were similar among the models, however, with a difference of approximately 10%. This directly influenced the better segmentation of the detected region, considering that the respective metrics; Sensitivity indicates a system’s ability to demonstrate the Injured area genuinely, and the Jaccard Index represents a system’s ability to accuse an Injured region as close to the real one as possible.

Visually it is possible to analyze the result of the segmented image represented by Figure 5. The “seed” is planted in the image (1) using our fully automatic method, using the Mask R model output-CNN adjusted to the Parzen window until the growth of the lung border in the image (3) Figure 5.

#### 5.2.2. Result Second Stage—(Step 2: Comparison with Literature Methods)

In this subsection, the Proposed Method results are presented compared to the literature methods already consolidated. Table 3, Table 4 and Table 5 shows the comparisons between classic works with excellent performances in different evaluation metrics. Tables 7 and 8 show comparisons with different current and innovative consolidated models in the literature, using deep learning and transfer learning-based methods with excellent results. All works used the same database, thus consolidating the experiments’ results in this study.

In Step 2, Table 3 presents the evaluation metrics, along with the methods in the literature in comparison to the model shown in this work, with the objective of segmentation in pulmonary CT images. Both works used the same database, containing 36 separate images from the data-set presented in this Section 4.1.

It can be seen that our model achieved better performance in all metrics. In the first column, we present the comparison methods, including the method proposed by this study. In the second, third, and fourth columns, the metrics are presented; Mcc, Acc, and Se. The proposed method surpassed all the methods presented in Table 3, reaching (98.34 ± 01.13) Accuracy (Acc), against HRV methods with (97.88 ± 01.29), GVF (96.79 ± 03.61) RHT mod (97.88 ± 01.29) and RHT multi com (95.77 ± 03.96). Accuracy is responsible for measuring the proportion of pixels that are correctly targeted. This means that the method with the greatest accuracy has the greatest ability to target a larger proportion of pixels in the lung region. The gain was also higher among the Mcc metrics reaching (93.72 ± 03.99), and Sen with (98.84 ± 00.97). This denotes that the metrics used, our model demonstrated superiority. The high result of the Mcc metric corresponds to greater efficiency, incorrectly predicting the lung region. Bearing in mind that the coefficient analyzes the association between the region indicated and the region given as true. In this way, both the non-pulmonary regions and the regions detected as pulmonary regions obtained satisfactory values in the evaluation metrics presented in the experiment.

**Figure 8:**
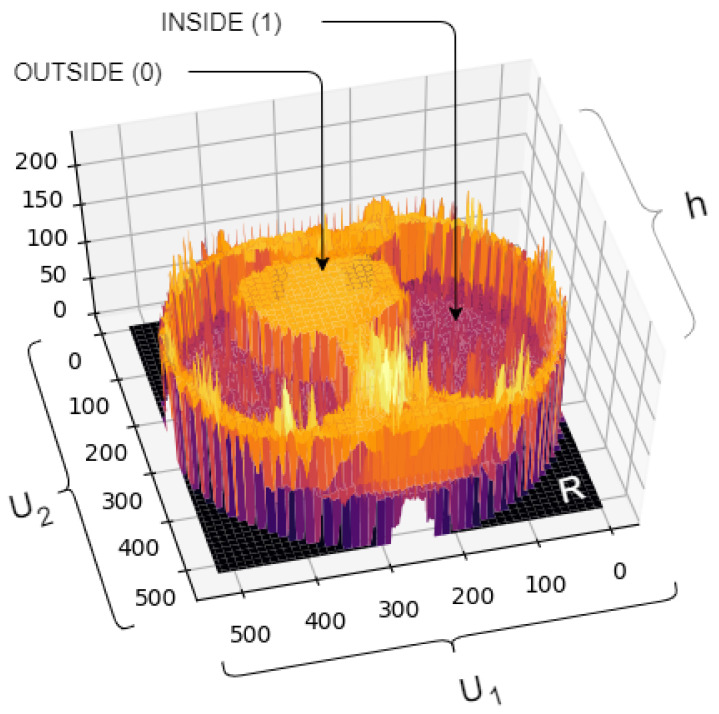
The Figure above shows the lung segmentation in 3D using our Proposed Method, based on deep learning in conjunction with the Parzen window method through transfer learning.

The Table 4 shows metrics related to the distance and overlap between images. The proposed method surpassed all models. We surpassed all models with values above (10.00) points of difference in the metric; Jaccard compared to the runner-up: Our offer (97.93± 01.64) against HRV (87.62± 04.81) This means that the region segmented by the model proposed in this study is more similar to the region of ground truth than the model in comparison. Furthermore, about the HD metric, our value reached (7.03 ± 00.28), indicating greater stability given the good values of approximation at zero and its deviation stoner. Figure 8 illustrates a 3D image of a pulmonary CT image segmentation with the proposed model. After the image is normalized to 0–255, the gray levels are shown as altitude in the image (axis h).

**Figure 9:**
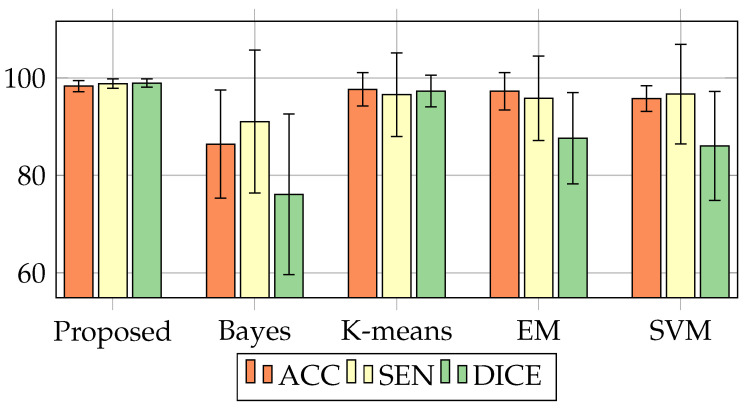
Illustration between metric values obtained, comparing the Proposed Method to other methods in the literature based on transfer learning presented by Table 7, with the combined models; Mask + Bayes, Mask + Kmeans, Mask + EM and Mask + SVM from the study of [13].

**Figure 10:**
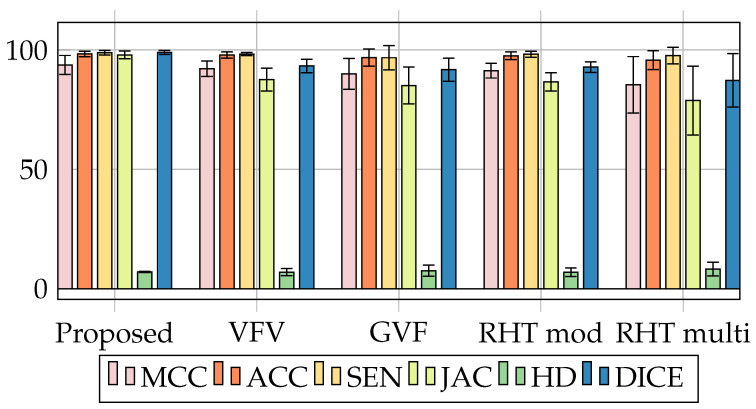
Illustration between metric values obtained compares the proposed method to other methods in the literature presented in Table 4.

This can be seen and better contextualized in Figure 9, represented according to Table 3 and Table 4. As can be seen in Figure 10, except for Hausdorff Distance, the metrics oscillate between 0 to 100%. The closer to 100, the better the method’s efficiency. However, with the Hausdorff metrics, the closer to 0, the more efficient the result.

**Figure 11:**
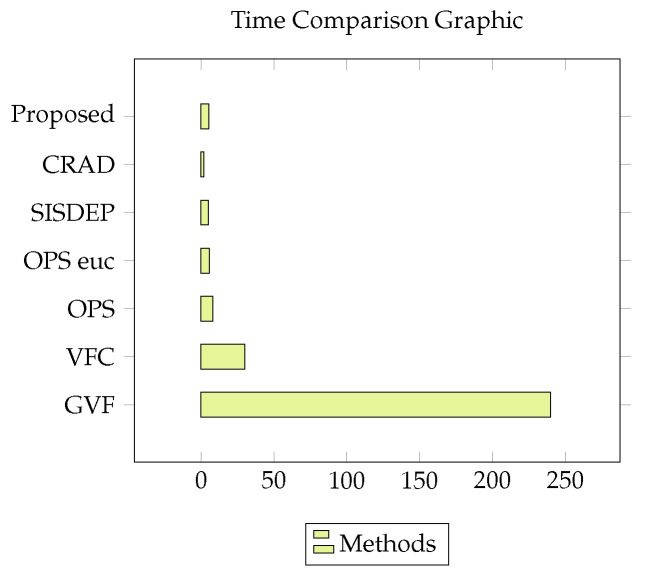
This Figure shows the segmentation time between models in the literature, compared to the method proposed by this study, the visual data refer to the Table 5.

As can be seen in Table 5, our proposed model achieved significantly good performance to the processing time compared to the models of the literature-based only on region growth. Despite using training with the convolution model, our application reached 5.43 s in time, surpassing and assimilating to methods already consolidated in state of the art such as GVF, VFC, OPS, and OPS Euclidean, Since all models have the same database in common in their experiments. Our method stands out among the models because it is a completely automatic method for segmenting the lung. This can be seen in Figure 11 the contrast between the segmentation time values between models found in the literature.

The statistical test, presented in the Table 6, was performed comparing the results of the proposed method to the other methods mentioned in work in the Table 3 and Table 4. The test obtained the result revealing itself to be statistically different between the other methods, thus proving their effectiveness and divergent characteristics. The symbol ◯ represents the difference, and Δ represents equality.

The Table 7 was added to validate our model among the methods using Mask R-CNN based on fine-tuning and transfer learning. As you can see, our best model surpassed all the models presented in [13], showing its effectiveness and better performance based on the results.

Combinations of methods for lung segmentation; Mask + SVM, Mask + Bayes, Mask + Kmeans, Mask + EM and Mask + SVM, contained in the work of Qinhua HU [13], were inferior to the results obtained by our method (Proposed Method), as shown in the Table through metrics; Acc com (98.34 ± 01.13), Sen (98.84 ± 00.97) and Dice reaching (98.95 ± 0.85). Compared to the best model proposed by Qinhua Hu Model (Mask + Kmeans) reached Acc (97.68 ± 3.42), Sen (96.58 ± 08.58) and (97.33 ± 3.24). Although the model had a satisfactory performance, standard deviation values among the three metrics of Qinhua Hu vary above 3%, reaching oscillate in 8.58%. In their worst model, the metrics oscillate with a difference above 12%, as is the case of the Mask + Bayes model for the Accuracy metric and its proportionality to the lung’s demarcation border.

According to the Table 8, our model outperformed (5.43 ± 00.21) s, against (11.24 ± 02.57) its best model, decreasing efficiently targeting time in half. The Proposed Model was superior in the process of classification and segmentation of the pulmonary region. Unlike the Mask + Kmeans model, our model used an IoT-based network to identify lung images in the CT scan image. This process was performed manually by the method performed in the work of [13]. This process is performed because, in some CT slices, no images of the lungs are found. This process identifies pulmonary regions to be segmented at CT images.

Thus, all the results presented and discussed were compared in different approaches. The steps discussed showed the Proposed Model’s excellent performance, contrasting other comparisons between classic and current models found in the literature for pulmonary segmentation.

## 6. Conclusions and Future Works

This work has as the premise, an innovative model based on excellent results. They were the results obtained discussed and compared to the current literature, thus presenting an excellent performance. We propose to this study a new approach with excellent results using deep learning for automatic segmentation of lung images in CT using IoT and Mask R-CNN deep learning methods combined with fine-tuning. The proposed model used API through the application (Lapisco Image Interface for the Development of Applications—LINDA), an IoT-based platform for the classification of medical images. The [52] model classified and segmented lung images with excellent results. Among the various classifiers, the Xception MLP model was the most effective among the other models used, reaching 98.42% accuracy, 99.52% Specificity.

Our model demonstrated better performance and robustness in all metrics. This means that the transfer learning process with results from Mask R-CNN along with the Parzen method provided excellent results for lung segmentation in CT images using fine-tuning. The difference between them to Accuracy reached more than 9%, (98.84%) against (89.96) of Mask R-CNN without transfer learning) demonstrating that our model surpassed Mark R-CNN itself. The method showed efficiency using the deep learning model with Parzen’s windowing method through transfer learning and fine-tuning using the Mask R-CNN result as a starting point for the Parzen method’s input image. The model adjusted to the edges of the lung, as we used the fine-tuning process (with the weights generated by Mask R-CNN), achieving excellent segmentation results with (98.34 ± 01.13) Accuracy and segmentation time in just 5.43 s, optimizing the time in half, against 11 s to the method with transfer learning, thus presenting excellent performance.

Also, our fully automatic method differs from many leading models that required the use of pre-processing methods, based on DIP, among other ways, to improve the quality of their results or to use semi-automatic models for the segmentation of lungs. Our model based on deep learning produced the best values based on the metrics: (Mcc, Acc, Sen, Jaccard, and DC) surpassing works consolidated by transfer learning techniques presented in this study. Thus, based on the results acquired in Section 5 Results and Discussion, it is possible to compare and conclude the effectiveness of the composite model presented by this study. The results support the conclusions at different stages of testing. Our model successfully surpassed the results generated by Mask R-CNN, as shown in the textual discussion of the analysis about Table 2. Followed by the results of comparison with works in the literature, presented and discussed in Section 5.2.2 Result Second Stage—(Step 2: Comparison with Literature Methods), where our model performed better the different methods; innovative methods, current methods, and classics methods.

Thus, as a contribution to this study, considering the need for fast and accurate pre-diagnoses, a high performance and speed tool was developed, whose main application is to assist in the detection and segmentation of pulmonary images in CT exams. The application uses an innovative tool based on IoT to transfer learning and fine-tuning in the computational process.

For future work, we intend to use other lung databases as further experiments and use to segment different types of CT images, such as; melanoma, mammograms, heart, using premise through fine-tuning and transfer learning. Future work will also aim to assess the model’s generalizability for different types of medical images.

## Figures and Tables

**Table 1:** The Table presents results of the combinations between deep attribute extractors and the classifiers in the test set after training for the classification of CT images in lung or non-lung. The metrics used to validate the first stage of our approach were Accuracy (ACC), Sensitivity (SEN), Precision (PREC), Specificity (SPE) and F1-Score. In all, 253 images representing the test set were classified.

Model	Classifier	ACC(%)	SEN(%)	PREC(%)	SPE(%)	F-Score(%)
InceptionResNetV2	Bayes	91.30	79.17	76.00	94.15	77.55
	MLP	96.05	90.00	90.00	97.54	90.00
	Nearest_Neighbors	94.07	84.31	86.00	96.53	85.15
	Random_Forest	95.26	80.65	100.00	100.00	89.29
	SVM_Linear	96.44	91.84	90.00	97.55	90.91
	SVM_Polynomial	96.84	90.38	94.00	98.51	92.16
	SVM_RBF	97.63	92.31	96.00	99.00	94.12
Xception	Bayes	85.38	53.52	90.48	97.80	67.26
	**MLP**	**98.42**	**93.18**	**97.62**	**99.52**	**95.35**
	Nearest_Neighbors	93.28	75.51	88.10	97.55	81.32
	Random_Forest	95.26	84.09	88.10	97.61	86.05
	SVM_Linear	98.02	89.36	100.00	100.00	94.38
	SVM_Polynomial	97.23	87.23	97.62	99.51	92.13
	SVM_RBF	97.23	85.71	100.00	100.00	92.31
NASNetMobile	Bayes	78.26	41.27	59.09	90.53	48.60
	MLP	94.07	75.44	97.73	99.49	85.15
	Nearest_Neighbors	95.26	83.33	90.91	98.05	86.96
	Random_Forest	94.07	93.94	70.45	94.09	80.52
	SVM_Linear	95.26	82.00	93.18	98.52	87.23
	SVM_Polynomial	95.65	83.67	93.18	98.53	88.17
	SVM_RBF	96.05	85.42	93.18	98.54	89.13
DenseNet201	Bayes	84.58	81.82	19.57	84.71	31.58
	MLP	97.23	93.33	91.30	98.08	92.31
	Nearest_Neighbors	95.65	84.31	93.48	98.51	88.66
	Random_Forest	91.30	96.15	54.35	90.75	69.44
	SVM_Linear	96.84	95.24	86.96	97.16	90.91
	SVM_Polynomial	96.84	95.24	86.96	97.16	90.91
	SVM_RBF	96.84	95.24	86.96	97.16	90.91
ResNet50	Bayes	83.79	59.18	58.00	89.71	58.59
	MLP	94.86	83.64	92.00	97.98	87.62
	Nearest_Neighbors	95.26	83.93	94.00	98.48	88.68
	Random_Forest	91.70	89.19	66.00	92.13	75.86
	SVM_Linear	96.84	95.65	88.00	97.10	91.67
	SVM_Polynomial	96.84	93.75	90.00	97.56	91.84
	SVM_RBF	97.23	92.16	94.00	98.51	93.07
InceptionV3	Bayes	85.38	52.54	77.50	95.36	62.63
	MLP	96.44	87.80	90.00	98.11	88.89
	Nearest_Neighbors	96.05	82.61	95.00	99.03	88.37
	Random_Forest	94.47	90.62	72.50	95.02	80.56
	SVM_Linear	96.84	86.36	95.00	99.04	90.48
	SVM_Polynomial	92.49	69.81	92.50	98.50	79.57
	SVM_RBF	97.63	94.74	90.00	98.14	92.31
VGG19	Bayes	85.38	50.00	5.41	85.94	9.76
	MLP	96.84	87.18	91.89	98.60	89.47
	Nearest_Neighbors	96.05	84.62	89.19	98.13	86.84
	Random_Forest	96.05	84.62	89.19	98.13	86.84
	SVM_Linear	96.84	82.22	100.00	100.00	90.24
	SVM_Polynomial	96.84	83.72	97.30	99.52	90.00
	SVM_RBF	96.84	96.77	81.08	96.85	88.24
DenseNet169	Bayes	83.79	0.00	0.00	83.79	0.00
	MLP	96.05	84.44	92.68	98.56	88.37
	Nearest_Neighbors	94.47	77.55	92.68	98.53	84.44
	Random_Forest	94.47	90.91	73.17	95.00	81.08
	SVM_Linear	96.84	88.37	92.68	98.57	90.48
	SVM_Polynomial	96.84	88.37	92.68	98.57	90.48
	SVM_RBF	96.84	88.37	92.68	98.57	90.48
MobileNet	Bayes	87.35	55.56	89.74	97.89	68.63
	MLP	97.23	86.36	97.44	99.52	91.57
	Nearest_Neighbors	95.26	80.00	92.31	98.56	85.71
	Random_Forest	96.05	83.72	92.31	98.57	87.80
	SVM_Linear	97.63	88.37	97.44	99.52	92.68
	SVM_Polynomial	97.23	90.00	92.31	98.59	91.14
	SVM_RBF	96.44	85.71	92.31	98.58	88.89

**Table 2:** Metrics of the proposed method, being Matthews Correlation coefficient (Mcc), Accuracy (Acc), Sensitivity (Se), Jaccard index (Jaccard), Hausdorff distance (HD), Dice coefficient (DICE) e Time.

Metrics	R-Mask CNN	Proposed Method
Mcc	58.03 ± 06.46	**93.72** ± **03.99**
Acc	89.96 ± 04.38	**98.34** ± **01.13**
Sen	88.03 ± 05.41	**98.84** ± **00.97**
Jaccard	87.69 ± 05.69	**97.93** ± **01.64**
HD	7.21 ± 0.24	**7.03** ± **00.28**
DICE	93.34 ± 3.38	**98.95** ± **00.85**

**Table 3:** Comparison of the method proposed by this study between models in the literature; VFC, GVF, RHT mod, and RHT multi.

Methods	Mcc	Acc	Sen
**Proposed Method**	**93.72** ± **03.99**	**98.34** ± **01.13**	**98.84** ± **00.97**
VFC	92.13 ± 03.20	97.88 ± 01.29	98.26 ± 00.66
GVF	90.00 ± 06.45	96.79 ± 03.61	96.75 ± 05.10
RHT mod	91.34 ± 03.09	97.56 ± 01.63	98.22 ± 01.28
RHT multi	85.38 ± 11.85	95.77 ± 03.96	97.68 ± 03.45

**Table 4:** Overlap and Distance metrics for different compared methods. Comparison between proposed method and literature works.

Methods	Jaccard	HD	DICE
**Proposed Method**	**97.93** ± **01.64**	7.03 ± 00.28	**98.95** ± **00.85**
VFC	87.62 ± 04.81	**6.92** ± **01.50**	93.33 ± 02.82
GVF	85.11 ± 07.71	7.55 ± 02.29	91.76 ± 04.84
RHT mod	86.65 ± 03.79	6.93 ± 01.79	92.81 ± 02.21
RHT multi	78.82 ± 14.43	8.23 ± 02.91	87.29 ± 11.18

**Table 5:** Time average and standard deviation of the methods.

Methods	Average Time (s)
GVF	240.000 ± 3.05
VFC	030.00 ± 2.67
OPS	008.27 ± 2.63
OPS Euclidean	005.86 ± 1.96
SISDEP	004.90 ± 2.02
CRAD	002.00 ± 0.16
**Proposed Method**	**005.43** ± **0.21**

**Table 6:** Table with the result of Friedman’s non parametric statistical test.

Methods	Mcc	Acc	Sen	Jaccard	HD	DICE
VFC	◯	◯	◯	◯	◯	◯
GVF	◯	◯	◯	◯	◯	◯
RHT mod	◯	◯	◯	◯	◯	◯
RHT multi	◯	◯	◯	◯	◯	◯

**Table 7:** Comparisons between methods in the literature that use the Mask R-CNN network based on transfer learning.

Methods	Metrics		
	Acc	Sen	DICE
**Proposed Method**	**98.34** ± **01.13**	**98.84** ± **00.97**	**98.95** ± **00.85**
Mask + Bayes	86.42 ± 11.11	91.06 ± 14.66	76.10 ± 16.49
Mask + K-means	97.68 ± 03.42	96.58 ± 08.58	97.33 ± 03.24
Mask + EM	97.28 ± 03.85	95.86 ± 08.67	87.63 ± 09.39
Mask + SVM	95.78 ± 02.62	96.69 ± 10.24	86.05 ± 11.21

**Table 8:** The segmentation time of our proposed model has compared to the [13] method.

Method	Second
**Proposed Method**	**05.43** ± **00.21**
Mask + K-means	11.24 ± 02.57
